# Human Behavior and Cognition in Evolutionary Economics

**DOI:** 10.1007/s13752-012-0036-4

**Published:** 2012-10-31

**Authors:** Richard R. Nelson

**Affiliations:** Columbia Earth Institute, Columbia University, New York, NY USA

**Keywords:** Bounded rationality, Innovation, Problem solving, Selection, Understanding

## Abstract

My brand of evolutionary economics recognizes, highlights, that modern economies are always in the process of changing, never fully at rest, with much of the energy coming from innovation. This perspective obviously draws a lot from Schumpeter. Continuing innovation, and the creative destruction that innovation engenders, is driving the system. There are winners and losers in the process, but generally the changes can be regarded as progress. The processes through which economic activity and performance evolve has a lot in common with evolution in biology. In particular, at any time the economy is marked by considerable variety, there are selection forces winnowing on that variety, but also continuing emergence of new ways of doing things and often economic actors. But there also are important differences from biological evolution. In particular, both innovation and selection are to a considerable degree purposive activities, often undertaken on the basis of relatively strong knowledge.

Before reflecting on the kind of theory of human behavior and cognition needed in my brand of evolutionary economics, I need to lay out the empirical and theoretical orientation of that body of research and writing. I say “my brand” because there are several different strands of economic theorizing that sometimes are called “evolutionary”; and this essay is focused on the kind of evolutionary economics that Sidney Winter and I are associated with (see, e.g., Nelson and Winter 1982, 2002). I will contrast this kind of evolutionary economics with those other brands later.

## The Orientation of Evolutionary Economics

I focus first on the subject matter addressed. My kind of evolutionary economics starts from the position that modern economic systems that make extensive use of for-profit firms competing for customers on relatively open markets are always in motion, always evolving, driven by the continuing innovation that this kind of an economic system induces. We would argue that the cumulatively vast increases in living standards and productivity experienced by a significant part of the world’s population are the most dramatic and beneficial achievement of the competitive, market-organized economies that began to emerge in the late eighteenth and early nineteenth centuries. Therefore, among the most important challenges for economic theory are to illuminate how this miracle was accomplished, the factors behind the differences across parts of the world in the extent to which they have participated in it, and the determinants of economic growth in the future.

More generally, like Marx and Schumpeter, evolutionary economists see innovation as a central aspect of the competitive process going on in many industries. As a result of continuing innovation, the economy always is evolving, always is in the process of transforming itself. Within industries, some firms rise and some decline. Totally new industries based on new technologies come into existence and older ones disappear. The uneven pace of technological change across product fields and industries is a principal cause of the major changes in the structure of prices, and in the allocation of labor among economic sectors, that have occurred over the years.

This is a very different view of what is most important about modern economic systems and how they work, than that of the standard economic analysis that now dominates the writings and teaching in academic economics departments. Within a standard contemporary economics text (e.g., Taylor 2008), considerable attention is indeed paid to economic growth, and it is recognized that technological innovation is the basic driver of growth. However, economic growth is treated as basically an “aggregate” phenomenon, measured by increases in real GNP per capita or per worker hour, with little attention to the major differences across industries in rates of productivity growth and other manifestations of uneven technological change. There is hardly any examination of how technological progress occurs, or the kind of competition that goes on in industries where innovation is important. The analysis of resource allocation and prices assumes an economy in equilibrium, not one marked by continuing uneven technological progress. Similar assumptions underlie the analysis of what competition is all about.

I note that the much more central focus of evolutionary economics on technical change and economic growth is not a new thing in economics. This is what Smith’s *The Wealth of Nations* [(1776)1937] is mostly about. Smith and the “classical” economists who followed him also were concerned with the determinants of prices and the allocation of resources. But these subjects generally were treated after the sources of economic growth had been discussed. Marx [(1867) 1932] also had economic growth at the center of his analytic attention, and his analysis of the structure of industry and of competition was part of his growth theory.

As the nineteenth century progressed, the treatises on economics paid less and less attention to economic growth, and focused more and more on market mechanisms and how they determined prices and the allocation of resources in an economic equilibrium. Marshall (1948) *Principles of Economics* has very little in it on economic progress. When in 1911 Schumpeter (1934) wrote his *The Theory of Economic Development*, a book that has had an enormous influence on the development of modern evolutionary economics, he clearly knew that he was swimming against the tide.

As I have said, the central focus of modern evolutionary economists on long-run economic growth certainly does not mean that we are not interested in what determines the configuration of prices that one finds in an economy at any time, or the allocation of resources to meet different kinds of wants and among different kinds of activities and economic sectors. However, like Smith we treat these subjects as being molded by the dynamics of economic growth. And like Marx and Schumpeter, we treat both the organization of industry, and the waves of depression and inflation that have marked the history of capitalist economic development, as integral aspects of the economic growth process, and not analyzable independently of understanding of what drives growth.

I note that, while economists of evolutionary persuasion have in mind a broad reform of economic analysis, to date our work has tended to cluster in three somewhat overlapping areas. One is study of technological change (for a survey see Dosi and Nelson 2010). A second is theorizing and associated empirical work on how business firms actually operate, and what determines their capabilities and competitive prowess (see Dosi et al. 2000). A third area in effect combines these two strands. It is concerned with the dynamics of Schumpeterian competition, and how the structure of firms and of the industry as a whole tend to co-evolve with technologies that are employed (Malerba et al. 1999). Recently the scope of research by evolutionary economists has been expanding, with forays into topics like consumer behavior, and economic institutions and their evolution.

While these different arenas and foci call for somewhat different structures of analysis, in all of them evolutionary economists highlight the often shifting ground on which economic agents stand. Some aspects of the environment in which they are operating may be relatively stable, in which case they can learn patterns of action, routines that continue to suffice to meet their objectives. On the other hand, other aspects of their environment, or the general context in which they operate, may have shifted in such a way as to make what they had been doing no longer effective in meeting their wants. When this is not recognized, past behaviors may continue, but the results may be unsatisfactory or worse. When the situation is recognized, the economic agent faces the challenge of trying to do something that is effective in a context which is unfamiliar and imperfectly, perhaps even wrongly, understood. In some contexts, or areas of action taking, change in the economic environment is sufficiently slow so that relatively modest periodic changes in routine suffice. In other contexts survival of the economic agents may require continuing innovation, and some luck as well as skill in identifying new things to be doing.

I want to turn now to another difference between the orientation of my brand of evolutionary economics and the general presumptions of my mother discipline that may be as or more fundamental than differences in how the economic system is viewed and the focal subject matter for study. It is regarding the nature and function of good “theory.”

I and my kin are for the most part “inductivists.” Of course since Hume’s time no serious scholar has been a simple, pure inductivist. However, some scientists believe that theorizing should proceed from paying close attention to the empirical phenomena being studied, identifying broad regular patterns, and then trying to develop reasons for these that also seem consistent with other empirical evidence. At the other end of the spectrum, many scientists believe that at least initial theorizing should be free to make hypotheses that are at some distance from what is known empirically, and that the business of empirical work is to calibrate or test theory.

It is fair to say that while the latter is the view of most economists today, the evolutionary economists I work with are largely in the former camp. We believe in looking closely at the phenomena we are studying with a relatively open mind, and then coming to an explanation for what we think we see that makes sense, and is consistent with other things we know empirically. Since for the most part theorizing in evolutionary economics involves specifying the processes that generate the phenomena to be explained, this is a commitment to “process realism,” in a sense I will develop later.

## In What Sense is Evolutionary Economics Evolutionary?

I hope I have made clear what my broad brand of evolutionary economics is about, and the view it holds regarding the kinds of phenomena that theory needs to address, and some of the characteristics of a good theory. Let me now try to explicate in what sense the economics I have described is “evolutionary.”

It is evolutionary, first of all, in the sense that its central focus is on change, and its explanation of why things are what they are at any particular moment of time involves centrally analysis of how they got that way. But second, to narrow the concept of evolution, the mechanism of change presumed by the theory involves the assumptions that at any time there exists a variety of versions of whatever it is that is in the process of changing, that there are mechanisms of selection that winnow systematically on this variety, favoring some versions and penalizing others, and that there also are forces at work that renew variety.

In developing our theoretical framework were we influenced by evolutionary theory in biology? Certainly we were, to some degree.

But our belief that economic change was an evolutionary process came to us largely by induction, by reflecting on the empirical phenomena we were observing, and the processes that we thought were generating those phenomena. I note that notions that social, economic, and political structures “evolve” in some sense long preceded Darwin and that, in a sense, modern evolutionary economists are returning to a line of social science theorizing that has been around for a long time (Hodgson 1993; Nelson 2006). Thus the common hallmark of the theories articulated in the eighteenth century by Mandeville on the evolution of the warship, Smith on the division of labor and economic organization, and Hume on political institutions, is the argument that the complex and sophisticated human-made structures that one can observe at any time were developed over a long period of time as a result of a series of incremental changes made by agents who at the time had particular objectives in mind, but who never foresaw what would be created over the long run from this cumulative process.

I also note that there are major differences between evolutionary economics and evolutionary biology regarding the particular mechanisms that are driving the evolutionary process. (For an extended discussion see Nelson 2006). One important difference is that human and organizational purpose and often sophisticated understanding play major roles in evolutionary economics. As I will elaborate later, evolutionary economics, at least our brand, draws heavily on Herbert Simon’s (1955) concept of bounded rationality. But while in comparison with more orthodox economics we emphasize the “bounded” aspects, we evolutionary economists do not see individual and organizational economic agents as like fruit flies. They often think about what they are doing. It is true that many animals often behave as if they were thinking. However, humans innovate deliberately. An important part of the innovation process involves thinking, doing research, calculating, operations going on in the mind.

A second difference is that the survival and continuity of economic agents is only occasionally at stake in the selection on economic practices. Sometimes it is, for example when competition in an industry is very strong. But often the individuals and organizations involved are able to change what they are doing so as to avoid duress. More generally, and connected to the point I made above about the role of cognition in economic evolution, it is common for individuals and organizations that are not under any particular pressure to adopt new ways of doing things because they have reason to believe that these would be improvements.

A third important difference is that many modern evolutionary economists, certainly myself included, highlight the crucial importance in economic evolution of the broad culture within which economic agents operate. In particular, while individual economic agents have particular beliefs and competences of their own, to a considerable extent how they think and what they are able to do is the result of their having grown up in, and operating in, a culture that includes their fellows. And the new things that individuals learn, or learn to do, become (if with a lag, and possibly in different form) part of the body of knowledge and technique on which their fellows can draw. As a consequence, the evolution of know-how and knowledge more generally needs to be understood as a collective process.

All three of these aspects sharply differentiate the way economic capabilities evolve from the processes involved in biological evolution. Let me make the argument concrete by considering how evolutionary economists treat technical change as an evolutionary process (for a survey see Dosi and Nelson 2010).

In virtually all fields of technology at any time one can observe a number of different agents investing the time and resources to try to come up with the design of a product or process that is better in some way from those currently in widespread use. These innovations are often in competition with each other, and with parts of prevailing practice. Some succeed and some fail. The winners and losers are sometimes determined by the market, sometimes by other selection mechanisms. And knowledge of what has succeeded and what has failed, as well as the nature of the winners and losers, becomes part of the environment for the next round of competitive efforts.

I note that evolutionary economists are not alone in adopting an evolutionary perspective on the processes through which human know-how and knowledge advance. It is interesting that the proposition that technological advance should be understood as an evolutionary process has been put forth by scholars studying the topic coming from a variety of different disciplines (see, e.g., Vincenti 1990). Today that language and analytic perspective enables communication and common understanding among scholars in the interdisciplinary community studying technological change.

Evolutionary economics treats a wide range of economic variables, not just technologies, as evolving. These include scientific knowledge, business organization and practice, the structure of industries, the allocation of resources in an economy, the institutions governing economic activity, and others. Some of the features of technological evolution carry over to these other arenas, but others do not. Indeed in my view an important challenge for evolutionary economists is to identify the differences in the evolutionary processes at work in these different spheres, as well as the common elements. However, I do not have space here to even sketch the differences.

## Different Brands of “Evolutionary” Economics

While this paper is about my brand of evolutionary economics, there are at least two other bodies of theorizing and writing about economic phenomena that sometimes have been called “evolutionary”: evolutionary game theory, and writing that aims to explain aspects of economic behavior on the basis of aspects of the human biological inheritance.

What is the relationship between my style of evolutionary economics and evolutionary game theory (see, e.g., Weibull 1995; Walliser 2012, this issue)? There is some overlap of subject matter addressed and modes of explanation. Both are concerned with change. Both see the change process as evolutionary in the sense that it involves variety and selection. However, the differences between the two camps strike me as at least as important as the similarities. As I proposed above, evolutionary economics is oriented towards a relatively well-defined body of empirical subject matter, and the “evolutionary” aspects of the field are invoked because they seem to explain that subject matter. In contrast, evolutionary game theory is largely defined by a general body of theory and technique, and the subject matter addressed is chosen largely because that body of technique seems applicable to it. And using the language I employed earlier, theorizing in evolutionary economics is largely inductive with (in good work within the tradition) the empirical subject matter addressed usually described in some detail. On the other hand, in evolutionary game theory, when empirical subject matter is addressed that subject matter generally is formulated in quite abstract form.

In recent years there has been a resurgence of interest in trying to explain some economic phenomena, particularly aspects of economic behavior, as having been influenced significantly by the evolved biological characteristics of modern humans. This strand of “evolutionary” economic analysis obviously has close kinship with the parts of sociobiology concerned with human behavior and society, and with evolutionary psychology (e.g., Crawford and Krebs 2002; Gandolfi et al. 2002; Frey and Stutzer 2007). I would propose that, while there is no essential incompatibility between this line of analysis and evolutionary economics as I have described the field, they are different endeavors. To date my brand of evolutionary economics has not found it necessary, or useful, to invoke the biological aspects of humankind in our theorizing about behavior and cognition. But it is possible that a certain amount of understanding could be gained by exploring this route.

## The Behavior and Cognition of Economic Agents in Evolutionary Economics

To get to the subject of the workshop, what is the view of the behavior and cognition of individuals and organizations contained explicitly or implicitly in our brand of evolutionary economic theory? Like the view on these matters that increasingly is coming to the fore in cognitive science, it is multifaceted (as discussed in Nelson and Nelson 2002; see also Nelson 2011).

First of all, a considerable portion of behavior is seen as relatively automatically induced by the context. Habits, routines, customs, play a major role within our kind of economic evolutionary theory. Some of these may involve highly sophisticated patterns of behavior that required considerable effort and time to learn, but once learned become more or less automatic. But second, another aspect of the behavior of economic agents, operative in different contexts, involves problem solving, exploring alternatives both physically and in the mind, discovering and inventing ways to meet a challenge. Third, while for the most part human and organizational problem solving proceeds in close interaction with a particular problem, humans also can step back from the particular action context they are in, and reflect on matters that might be relevant more generally. Such reflection generally involves mental models. Fourth, these mental models, these beliefs that guide problem solving and efforts at inventing (and which also often rationalize prevailing ways of doing things) are seen as, to a considerable extent, drawn from aspects of collective culture. These aspects of collective culture may include the understandings of different fields of science, widely held notions about good business practice, and various ideological views common in the culture.

I think it useful to compare the orientation to theorizing about human behavior and cognition in evolutionary economics with the orientation in what has come to be called behavioral economics. (For a survey see DellaVigna 2009.) Both strands of heterodox economics harbor the belief that standard neoclassical theory is an inadequate theory of human behavior. Modern behavioral economics, as that subject has developed to date, has been mostly concerned with identifying aspects of human behavior, wherever these may occur, where the basic cannons of rational behavior as economists have defined that seem especially problematic. In contrast, in evolutionary economics the focus is on a particular body of empirical phenomena and the theorizing about human behavior and cognition is induced by the need to characterize and explain the actions of the involved human agents, particularly in the generation of and response to economic change.

I note also that, to date at least, the view of human cognition and behavior in behavioral economics has repressed the broad common cultural influences on individuals. For that reason the theorizing in evolutionary economics may have more in common with that in old-style institutional economics, which highlighted the collective cultural influences on human beliefs and behaviors (as described in Hodgson 1999).

I now want to make these somewhat general observations about the treatment of individual and organizational behavior and cognition in evolutionary economics more concrete by focusing on two important topics where as I noted there has been substantial research and theorizing within the tradition: technological advance, and firm capabilities and behavior.

It seems clear that the processes involved in technological advance involve all the aspects of human behavior and cognition that I listed above. There is, first of all, a significant amount of routine involved in or connected with inventive work, particularly if that work is done by a team of persons employed by a large organization. Records need to be kept of what was accomplished when, among other reasons to enable patent applications to be defended, orders for materials written up and put in the works, meetings attended, reports given, etc. But the cutting edge of the work is problem solving. This involves envisaging possible designs, conceiving ways to test out ideas, reflection on the factors behind what has succeeded and failed, and deciding how to proceed from here.

The concept of a “technological paradigm” (Dosi 1982) has played a major role in theorizing about technological advance. A technological paradigm is a body of understanding, technique, and problem-solving heuristics shared by professionals in a field. The prevailing paradigm shapes, constrains, and gives power to efforts to advance a technology. However, each individual or team aiming to do so brings to the task some beliefs, understandings, and experiences unique to it.

Let me turn now to firm behavior. From its beginnings, our brand of evolutionary economics has harbored the goal of building a theory of the firm that explained the things firms did that were of interest to economists in a way that was consistent with what was known about actual decision processes in firms. Several important empirical studies had shown that the processes by which firms determined variables like the prices they charged, orders for the inputs they used, and R&D expenditures, usually did not involve any explicit maximizing calculations, but rather often seemed largely to involve the use of relatively mechanical rules and routines. (One of the first and most influential of these studies was by Hall and Hitch 1939.) A significant community of scholars and a large body of research now has grown up around a theory of the firm in which it is assumed that firm behavior is largely determined by the routines it is using at any time. (Cyert and March 1963 is the original source of much of this work.) In the standard course of business operations, established routines are carried out without much in the way of conscious thinking about what should be done.

On the other hand, firms are not locked into their prevailing routines, and in many cases their continuing survival requires that they change what they are doing in response to changes in their competitive environment, and do so in a timely fashion. Another important part of the evolutionary theory of the firm, as it has developed, is concerned with what turns on the firm’s mechanisms for actively deliberating what it should be doing, and the changes it needs to make in its operating routines, and the nature and effectiveness of these deliberation processes.

A firm may be able to change particular things it is doing without any change in its broad orientation or strategy. On the other hand, strategy making and remaking also is an important component of the behavioral theory of the firm, and are activities assumed to involve conscious deliberation. In contrast with the carrying out of established routines, and the making of moderate changes in these, which is going on all the time, established strategies tend to be subject to significant revision only occasionally. The change process generally involves discussion among a small group of top executives. A number of alternatives may be considered, and then winnowed. Some explicit research may be involved in the process. However, several detailed studies (e.g., Leonard-Barton 1992; Tripsis and Gavetti 2000) have shown convincingly that the alternatives considered and the winnowing process are shaped to a considerable degree by the past experiences of the participants and by the then-fashionable beliefs about what makes for good business practice.

Not surprisingly a good share of the work of evolutionary economists on the theory of the firm has been concerned with understanding what is involved when firms innovate, the nature of the capabilities for timely and effective innovation that leading firms have, and of what is involved when lagging firms try to catch up. Again, an adequate characterization of what is going on in these cases would seem to require all of the kinds of behavior that I laid out earlier. And the analyses here clearly blur into the writings on technological change more generally.

I noted earlier that evolutionary economists are beginning to study consumer behavior. Let me consider now the kinds of behavior assumed in my article with Davide Consoli on “An Evolutionary Theory of Household Consumption Behavior” (Nelson and Consoli 2010). The variegated view of individual behavior and cognition that one sees in the evolutionary perspective on how technology progresses and on firm behavior shows up again in this apparently very different context.

Household consumption behavior is seen as to a large extent a matter of acquired habits and routines. But there are occasions when conscious decision making, including some research, is involved, in particular for big-ticket items like the purchase of a new automobile or the choice of a college for one’s children. And in both of these cases the individual consumer or household needs to be understood as a member of a broader community, and strongly influenced by the culture of that community, which molds their preferences, their routines, and how they think about alternative courses of action.

And there are occasions in which households, like firms, are required to “innovate.” Not surprisingly we evolutionary economists are particularly interested in how consumers respond to new goods and services. Here, they may have little experience of their own to draw on, and when the good is first introduced to the market there is no cultural experience on which to draw. Particularly for new goods that have capabilities and qualities that established goods do not have, households have to make judgments as to what needs the new good might meet, and whether meeting those needs is worth the price. Some experimentation may be required before the questions are answered.

After some households adopt a good, notions about what the good is for enter the culture. And we enter the realm of diffusion theory.

## The Challenge for Cognitive Science

The treatment, or rather the treatments, of human cognition and behavior in evolutionary economics clearly are very different from how these subjects are treated in neoclassical economics. In the first place, evolutionary economics is committed to trying to “get the process right.” The presumption is that understanding of how human actions are generated in particular contexts is an essential part of a theory that aims to explain or predict what those actions are. The argument in neoclassical theory that economic agents behave “as if” they maximized utility, and it does not matter how they actually arrive at the actions they do, is not acceptable from this point of view.

Second, in many of the contexts that are of interest to evolutionary economics there is no way that the economic agents can have a full understanding of all the actions that are possible and the consequences of each, nor has there been sufficient experience for them to arrive at the best action through trial and error learning. In many contexts it is clear that a number of economic agents are making mistakes, in the sense that they are being stimulated to change what they are doing as the consequences become clearer, And in these and other contexts it is typical to see economic agents, who have similar goals and face similar constraints, doing different things, and getting different results.

As noted, Herbert Simon’s (1955) conception of “bounded” rationality fits much better the view of human goal-oriented activity contained in evolutionary economics than does the full-blown rationality of neoclassical economics. The concept does not deny the role of purpose and reflection in guiding human action, but highlights the limits of human understanding. The proposition, also argued by Simon and his colleagues, that much of goal-oriented behavior involves the following of established routines similarly does not deny purpose and intelligence, but rather highlights both that the actions engaged in today are ones that seemed to work satisfactorily in the past, and that there are real costs involved in continuingly rethinking what to do (March and Simon 1958; Cyert and March 1963). Both of these conceptions clearly have had a major influence on evolutionary economics.

So have developments in cognitive science. Scholars in cognitive science increasingly are recognizing—highlighting may be a better term—that humans get things done and respond to stimuli in a variety of different ways, depending on what they are doing and the circumstances.

For a long time the focus of cognitive science was on human problem solving that required, or appeared to require, conscious cognition. The artificial intelligence branch of cognitive science, which was dominant in the early days of the new field, assumed that the process of human problem solving involved information processing in a manner similar to that built into computer programs. But from the field’s beginnings there have been strong objections to the “humans think like computers operate” point of view. (For a brief historical discussion see Nelson and Nelson 2002.) One position that developed in opposition argued that “pattern recognition” should be understood as the central element of human problem solving, and in the responses of humans to circumstances that occur and require a response. While these two camps in computer science still stand mostly at odds, in my view the processes of inventing and strategizing—important elements of evolutionary economics—often tend to involve both elements. And the debate in cognitive science has helped me to recognize this.

There also has emerged within parts of the cognitive science community recognition, and insistence, that much of human action taking occurs relatively automatically (through the implementation of “routines”), although the pattern of action may be the result of considerable learning from earlier experiences (see, e.g., Hendriks-Jansen 1996; Hutchins 1996). In some cases this line of argument is linked to analysis that stresses the importance of pattern recognition as a trigger for particular action, treating pattern recognition as something humans do often without much thinking about it. But more generally, the realm of conscious deliberation, which long had been the focus of scholars in the field, now is recognized by many as comprising an important part of human behavior, but only a part. Merlin Donald (1991) in particular has stressed the varied nature of human activity and the cognitive processes involved.

In view of Herbert Simon’s role in the shaping of cognitive science, it is interesting that at least two of his earlier conceptions regarding human cognition and behavior are largely repressed in the field. One is the basic argument behind the concept of bounded rationality that in many contexts no amount of thought, calculation, and study can yield the absolute best solution to a problem. It is not even clear that a best solution can be defined, except tautologically. And while it may be helpful in such circumstances to try to identify earlier problems that were similar in some way, often there will not be any close match. Yet this is exactly the context within which many efforts to innovate proceed. To date cognitive science has not dealt much with this kind of problem solving.

The second is his recognition that much of human cognition and action taking takes place as part of a team, as part of a collaborative effort to do something. While his 1958 book with James March, *Organizations*, is about behavior in and of formal organizations, even when formal organization is not the context, much of human activity involves doing things with other people. It is clear that in such contexts such matters as shared language, and shared understandings of what the goal is and of how to achieve it, are crucial elements of the human cognition that shapes behavior. With few exceptions (see, e.g., Donald 1991; Hutchins 1996) these matters are beyond the scope of contemporary cognitive science.

More generally, while there is promise in some recent developments, there still is limited recognition among most scholars working in the field of cognitive science of the role that a shared culture plays in molding human behavior and cognition. When an engineer begins to analyze the problem of, say, how to improve the fuel efficiency of an engine, he or she brings to the table not just his or her past experience in wrestling with problems like this one, but a good share of the accumulated knowledge of generations of technologists and scientists that is relevant to the current problem. Cognitive science presently is largely blind to this. Ignoring the influences on human cognition and behavior that come from the culture individuals grow up in and live in may be legitimate given the way cognitive science has defined its tasks and goals. But evolutionary economists cannot ignore this, because what they are studying is exactly the processes through which important elements of human culture evolve.
